# Synthesis of Bio-Based Polyester Resins for Vat Photopolymerization 3D Printing

**DOI:** 10.3390/ma17081890

**Published:** 2024-04-19

**Authors:** Ines Cazin, Martin Ocepek, Janez Kecelj, Aleš Stanislav Stražar, Sandra Schlögl

**Affiliations:** 1 Polymer Competence Center Leoben GmbH, Sauraugasse 1, A-8700 Leoben, Austria; ines.cazin@pccl.at; 2 Helios Resins, Količevo 65, 1230 Domžale, Slovenia; martin.ocepek@resinshelios.com (M.O.); janez.kecelj@resinshelios.com (J.K.); alesstanislav.strazar@resinshelios.com (A.S.S.)

**Keywords:** additive manufacturing, vat photopolymerization 3D printing, bio-based polymers, photocuring, polyester resins

## Abstract

Driven by environmental considerations, the scientific community has directed great effort towards the synthesis of new materials derived from renewable resources. However, for photocurable resins, most commercially available building blocks still rely on petroleum-based precursors. Herein, we present a simple synthesis route for bio-based acrylate-modified polyester resins, whose viscosity is sufficiently low for processing them with vat photopolymerization 3D printing. The established synthesis route enables the gradual substitution of fossil-based raw materials with bio-based alternatives. The acid number, color and viscosity of the bio-based acrylic resins are characterized and photocurable formulations are prepared by adding a radical photoinitiator. The photopolymerization kinetics, and thermomechanical and mechanical properties of the photopolymers are investigated as a function of the resin structure and benchmarked against a commercially available petroleum-based counterpart. Finally, the processability of the new bio-based resins via digital light processing 3D printing is demonstrated and test specimens are successfully 3D printed with a resolution in the millimeter range.

## 1. Introduction

Additive manufacturing (AM), or three-dimensional (3D) printing, is a technique that offers the design freedom of 3D objects with high complexity and diverse properties [1]. Virtually designed 3D models can be developed into an object by using different processes of AM, such as extrusion, direct energy deposition, powder solidification, sheet lamination and photopolymerization [2]. The interest in AM is steadily growing, and by the end of 2029, the worldwide revenues for materials produced by AM techniques are forecast to grow to USD 23 billion in the market shares of automotive, defense, aerospace, medical and dental industries [3]. Polymers comprise the largest fraction of these produced materials [4]. Conventional microfabrication techniques such as replication methods, photolithography and subtractive processes are typical time-consuming operating procedures, which require tedious assembly and bonding to make multi-layered three-dimensional objects. In contrast, 3D-printing is based on a layer-by-layer approach that creates an object directly converted from computer-aided design to hardware. Complex 3D objects are printed without any need for bonding or alignment during the printing process [5,6]. Among the several polymeric AM methods, digital light processing (DLP) 3D printing has attracted significant attention over the past decade, due to the possibility of producing complex-shaped objects and its high flexibility [7]. As an optical 3D printing technology, DLP is based on a light beam, which is focused on the bottom surface of a vat filled with a photocurable resin. Due to local solidification, the desired geometry is constructed layer by layer [8]. With the DLP process, it is possible to reach a precision of tens of micrometers in the x-, y- and z-directions, and to print objects with high surface quality [9,10]. Despite the high precision that can be achieved, due to the limited size of the projection, only small-sized objects can be printed, which limits the applications in some fields [11]. Moreover, the technique requires a photoreactive component which limits the choice of material, while the viscosity of the resin should be in the range of 0.25 to 5 Pa·s. A fast curing speed is important to ensure a quick build speed of the object. In addition, a slow curing speed can cause printing defects such as warping, sagging or layer misalignment.

Based on the previously described advantages, it is not surprising that applications for DLP 3D-printed objects continue to grow rapidly and range from soft robotics, sensors and wearable electronics to biomimetic devices [12,13].

For DLP 3D printing, photocurable resins are required, which typically contain (meth)acrylate or multifunctional epoxy monomers/oligomers. Local solidification of the resin is obtained by light-triggered polymerization and curing reactions. (Meth)acrylate resins are cured via a radical chain-growth polymerization, while epoxy resins are polymerized in a step-growth manner following a cationic mechanism [14]. However, the majority of commercial photocurable resins are derived from non-renewable building blocks [15]. Implementing bio-resins for DLP 3D printing offers many ecological and economic benefits, such as high biodegradability and low toxicity, multiple recycling options and reduction of greenhouse gas emissions [16].

Thus, in the last few years, great effort has been devoted to the development of 3D-printable resins that are replacing petroleum-derived precursors with materials derived from renewable resources [3]. Some reported renewable building blocks include lignin, rosin and various carbohydrates [17,18,19]. The most accessible and non-expensive choice is plant oils (e.g., soybean, castor and linseed oil), which can be easily converted into UV-curable oligomers across their triglyceride structure [20,21,22,23]. One of the most promising derivatives from this category is castor oil, which has been used in the synthesis of UV-curable polyurethanes. The natural hydroxyl groups of castor oil undergo polyaddition with isocyanates, resulting in the formation of hard segments that yield polymers with excellent mechanical strength, while superior flexibility is achieved by the long flexible fatty acid structures of castor oil [24,25,26]. However, the long aliphatic chain of plant-based oils compromise on material properties, and polymers often suffer from low glass transition temperature (*T_g_*), which limits their technical applications. Terpene monomers are another reported renewable source, which can be cured by light-induced thiol–ene chemistry [27]. However, the printed photopolymers also suffer from a low *T_g_* [28].

Recently, research on bio-based unsaturated polyester resins (UPRs) has become popular due to their low cost, simple curing process, good balance of durability and mechanical properties [29,30]. UPRs generally consist of unsaturated polyester (UP) and a free-radical polymerizable diluent such as styrene. Several studies describe the decrease and replacement of styrene by reactive diluents derived from less toxic and/or bio-based materials [31,32]. UPRs are typically obtained by the polycondensation of diols and unsaturated dicarboxylic acids or anhydrides, which can be taken from renewable feedstocks [33]. Prominent bio-based feedstock for UPRs include acids such as sebacic acid, succinic acid, adipic acid, gluconic acid, itaconic acid or levulinic acid, and glycols (1,3-propanediol, isosorbide, 1,4-butanediol, sorbitol, ethylene glycol, etc.) [34,35,36,37,38,39].

While bio-based polymers often suffer from poor mechanical properties [40], several publications reported on UPRs derived from itaconic acid that benefit from both a high bio-based content and improved mechanical properties [41,42,43,44,45,46,47,48,49]. In this context, in the last few years, there has been a growing interest in the use of itaconic acid, which is composed of two carboxylic acid functionalities, an α,β-unsaturated double bond and two carboxyl groups, which make it highly flexible for UPR chemistry. Itaconic acid was first synthesized by the thermal decarboxylation of citric acid in 1837 [41,50]. Today, it is produced on an industrial scale via fermentation with *Aspergillus terreus*, using glucose as a carbon source [51].

Recently, Čuk et al. replaced raw materials of synthetic polyester polyol with their bio-based alternatives and developed a synthetic route for a fully bio-based and solvent-free version of polyester polyol which can be used in combination with acrylic polyol for high-solid two-component polyurethane protective coatings for metal surfaces. The synthesized bio-based polyester polyol shows a high potential in the coating industry, where the properties are comparable to the properties of synthetic counterparts [52].

Herein, we present the synthesis and characterization of photocurable bio-based polyester resins for their use in 3D printing with vat photopolymerization. Using commercially synthetic polyester acrylate (PEA) as a synthetic benchmark, monomeric building blocks are step-wise replaced with bio-based alternatives such as 1,3-propanediol, sebacic acid, succinic acid, isosorbide and itaconic acid. In a comprehensive way, the influence of the building blocks on the molecular weight, viscosity and cure kinetics of the polyester resins are studied to assess their applicability for 3D printing. In this work, bio-based resins showed fast curing speed, which enables printing of 3D objects with an acceptable resolution for applications in several fields, e.g., printing of medical devices.

## 2. Materials and Methods

### 2.1. Materials and Chemicals

For the synthesis, the following monomers were used: succinic acid (99.5%) from Roquette (Lestrem, France), sebacic acid (99.5%) from Arkem (Colombes, France), 1,3-propanediol (99.9%) from Covation Biomaterials (Newark, DE, USA), cyclohexandimethanol (Eastman, Kingsport, TN, USA 98.5%), acrylic acid (Arkema, Colombes, France, 99.5%), itaconic acid (Novasol Chemicals, Kraainem, The Netherlands, 99.7%), isosorbide (Ecogreen Oleochemicals, Singapore, 99%), hypophosphorous acid, 2,6-di-tert-butyl-4-methylphenol (99.8% Oxiris Chemicals, S.A., Barcelona, Spain) and 4-methoxyphenol (HQMME) (98+%, BASF) and phthalic acid anhydride (Atmosa Petrochemie GmbH, Vienna, Austria 99.7%). Phenylbis(2,4,6-trimethylbenzoyl) phosphine oxide (Irgacure 819) was used as a radical photoinitiator supplied by Sigma Aldrich (St. Louis, MO, USA). Sudan II was obtained from TCI chemicals (Tokyo, Japan). All chemicals were used without further purification.

### 2.2. Polyester Synthesis

All bio-based polyester resins were synthesized in two steps. For the first step, the following materials were used:

- PEA-BIO-1: cyclohexanedimethanol, 1,3 propanediol, sebacic acid, succinic acid, hypophosphorous acid, phthalic acid anhydride.
- PEA-BIO-2 and PEA-BIO-3: isosorbide, 1,3 propanediol, sebacic acid, succinic acid, hypophosphorous acid, phthalic acid anhydride.

The listed materials were directly introduced into a 6 L three-necked glass reactor equipped with a mechanical stirrer, thermometer, condenser, water trap, nitrogen inlet and external heating. The reactor was charged with raw materials and the system was heated to 210 °C under constant nitrogen flux. Toluene was used as reflux solvent for water removal. The progress of the reaction was monitored by acid value titration until the acid value was below 3 mg KOH/g. Toluene was removed using a vacuum. The resin was cooled to room temperature.

The second step included the addition of acrylic acid (for PEA-BIO-1 and PEA-BIO-2) and itaconic acid (PEA-BIO-3) and the addition of 2,6-di-tert-butyl-4-methylphenol and HQMME. The system was heated to 120 °C under constant oxygen flux until the reaction mixture reached an acid value of around 10. The mixture was cooled to room temperature and filtered through a 190 µm filter.

### 2.3. Preparation of Resin Formulations

Photocurable formulations were prepared by mixing the respective resin with 3 wt.% of Irgacure 809 and 0.01 wt.% Sudan II. The formulations were homogenized with a vortex mixture two times (1 min at room temperature) until all components were dissolved.

### 2.4. Resin Characterization

The viscosity of the prepared resins was measured by using a modular compact rheometer MCR 102 from Anton Paar (Graz, Austria) with a CP/PP 7 plate. Each measurement was carried out with 1 mL resin at room temperature and a shear rate of 300 s^−1^.

The curing kinetics of the prepared resins were studied by Fourier-transform infrared spectroscopy (FTIR) on a PerkinElmer Spectrum One spectrometer (Hopkinton, MA, USA). All spectra were taken in transmittance mode over a wavenumber range of 4000 to 800 cm^−1^. Spectra were accumulated from 16 scans at a resolution of 4 cm^−1^ and the absorption peak areas were calculated with OPUS software (version 2.0). All samples were prepared by drop casting of 1.5 µL of resin between two CaF_2_ discs. The samples were then irradiated with an LED curing lamp (405 nm, Opsytec Dr. Gröbel, Ettlingen, Germany) at 8 mW/cm^2^. The conversion of acrylate functional group was calculated with OPUS software by evaluating the decrease in the characteristic IR absorption band at 1620–1636 cm^−1^. The degree of conversion (DC) was calculated from the following equation:

(1) $$\mathrm{DC}\%=[1-(\frac{C_{Peak}/C_{Reference}}{U_{Peak}/U_{Reference}})]\cdot 100$$

where ***C_peak_*** corresponds to the absorption area of the observed band of the cured sample and ***C_reference_*** to its reference peak. ***U_peak_*** and ***U_reference_*** are the absorption area of the relevant band and reference peak of the uncured sample, respectively.

Gel permeation chromatography (GPC) was performed on a Knauer Azura GPC (Berlin, Germany) system equipped with a differential refractive detector and an AppliChrom StyDiViBe-P 5 µm columns. The polymer solution (7 mg mL^−1^, 100 μL) was injected into the GPC system using tetrahydrofuran eluent at a flow rate of 1 mL min^−1^.

^1^H-NMR spectra were recorded on a Bruker (300 MHz) spectrometer (Billerica, MA, USA) using DMSO-*d*_6_ as the solvent. ^1^H-NMR shifts of synthesized polymers are reported in ppm (δ) downfield from tetramethylsilane (TMS) and were determined by referring to the solvent peak DMSO-*d*_6_ for hydrogen atoms.

### 2.5. Color Measurement

The color of the prepared polyesters was measured according to the SIST EN ISO 6271 standard using the spectroscopic method [53]. Measurements were performed on a Hach Lico 690 spectral colorimeter (Loveland, CO, USA). The resins were placed in a glass cuvette which was inserted into the measuring instrument. The color value was read from the screen of the instrument according to the platinum-cobalt (Hazen) scale.

### 2.6. DLP 3D Printing

The samples obtained from PEA, PEA-BIO-1 and PEA-BIO-2 were printed with an Anycubic Photo Mono S printer (Shenzhen, China) using a 405 nm LED as the light source. Then, 0.01 wt.% of Sudan II was added and the formulation was homogenized with a vortex mixer two times (1 min at room temperature) until the components were dissolved. The printing parameters are presented in Table 1.

PEA-BIO-3 was prepared in the same way as the previously described resins but DLP 3D printing was performed on a Doppio printer operating at 405 nm (intensity was 30 mW/cm^2^) manufactured by way2production (Vienna, Austria). The resin vat was heated at 50 °C during the printing process. The objects were printed with three different bottom exposure layer times: 100, 75 and 50 s.

### 2.7. Characterization of Mechanical and Thermomechanical Properties

The mechanical properties were characterized by a ZwickRoell (Ulm, Germany) Z1.0b static materials testing machine with a crosshead speed of 250 mm min^−1^. The dimensions of the DLP 3D-printed tensile specimens were 75 mm × 12.5 mm × 2 mm.

Dynamic mechanical analysis (DMA) was carried out on a Mettler Toledo (Greifensee, Switzerland) DMA/SDTA861e analyzer. Mechanical loss factors (*tan δ*) and storage moduli (*E′*) were monitored over a temperature range from −45 to 100 °C at a heating rate of 3 °C/min. The glass transition temperature (*T_g_*) was determined by the temperature at the maximum of the loss factor. For sample preparation, test specimens (30 mm × 4 mm × 1 mm) were fabricated by an Anycubic printer (Shenzhen, China). The printing conditions are shown in Table 1.

## 3. Results and Discussion

### 3.1. Polyester Synthesis

The main goal of the research was to develop bio-based acrylate-functional polyester resins as a greener alternative to commercially available derivatives. For this study, a commercially available synthetic resin PEA was used as a benchmark, which is derived from 1,6-hexanediol, cyclohexanedimethanol, adipic and acrylic acid. Three different resins were synthesized by selectively replacing the components of PEA with bio-based alcohols and carboxylic acids as building blocks (composition is shown in Table 2).

**Figure 1:**
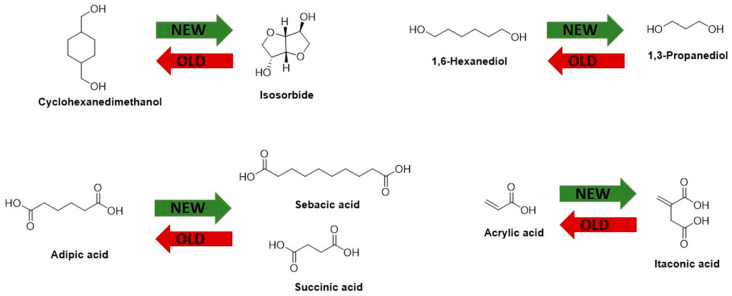
Representation of monomer structures.

In PEA-BIO-1, adipic acid was replaced with sebacic and succinic acid, and 1,6-hexanediol was replaced with 1,3-propanediol. In PEA-BIO-2, isosorbide was introduced instead of cyclohexanedimethanol, and in PEA-BIO-3, acrylic acid was additionally replaced by itaconic acid, yielding a polyester with the highest bio-based content (Figure 1).

**Figure 2:**
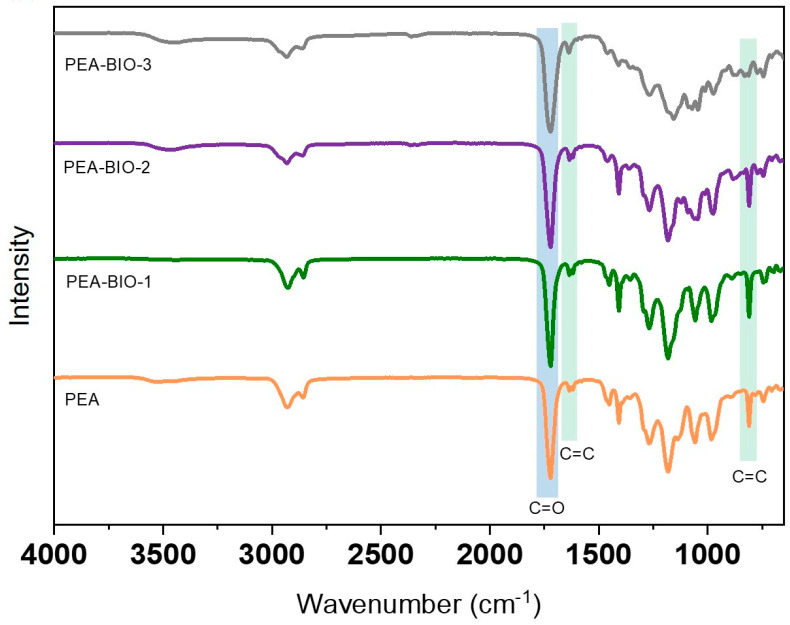
ATR FTIR spectra of synthesized PEA-BIO resins and the commercially available PEA resin.

The prepared acrylate polyesters were further characterized by ATR Fourier-transform infrared (FTIR) spectroscopy, and the most important vibrations are marked in Figure 2. The signals at 1619–1635 and 810 cm^−1^ correspond to C=C stretch and C=C deformation vibrations of the carbon double bonds, while the signal at 1730 cm^−1^ is assigned to the C=O stretching vibration of the ester group. The broad signal at 3500 cm^−1^ is related to the -OH groups of free carboxylic acid moieties [54,55,56].

The ^1^H-NMR spectra of the synthetic and bio-based resins are shown in Figure S1 in the Supporting Information. From these spectra, successful polymerization can be confirmed by the observed peaks around 4.0–4.3 ppm (highlighted in blue) which correspond to the protons of C(O)OCH_2_- groups. The protons assigned to the -CH_2_- group of 1,3-propanediol are observed between 1.5 and 2.5 ppm. The resonance signal at 2.6 ppm belongs to the -OC(O)CH_2_- group of succinic acid, while the corresponding peak of sebacic acid is located around 2.3 ppm. Two signals at 1.3 and 1.6 ppm correspond to the methylene groups of sebacic acid.

The average molecular weight (*M*_w_) of the synthetic and bio-based resins was analyzed by gel permeation chromatography (GPC) and the spectra are provided in the Supporting Information (Figure S3). The determined values are as follows: 1543 g/mol (PEA), 936 g/mol (PEA-BIO-1), 665 g/mol (PEA-BIO-2) and 1001 g/mol (PEA-BIO-3).

It was also found that the replacement of adipic acid (PEA) with sebacic and succinic acid (PEA-BIO-1) resulted in a decrease in the viscosity from 2.31 Pa·s to 0.27 Pa·s. By introducing isosorbide into the structure (PEA-BIO-2), the viscosity increases to 0.96 Pa·s. This can be explained by the cyclic and relatively rigid structure of isosorbide, which makes the related polymer chains less flexible and therefore causes an increase in viscosity. In PEA-BIO-3, the acrylic acid was additionally replaced with itaconic acid as a green alternative, which resulted in a significantly higher viscosity of 91.78 Pa·s. Compared to acrylic acid, itaconic acid contains two carboxylic acid groups which can cause increased viscosity through a higher number of hydrogen bonding and intermolecular interactions. It should be noted that for DLP 3D printing, the viscosity of PEA-BIO-3 was reduced by adding a bio-based reactive diluent. In particular, acrylated eugenol (AEUG) was used in a mass ratio of 1:1, yielding a viscosity of 0.36 Pa·s.

Higher molecular weight often leads to higher viscosity due to increased chain entanglements, resulting in more resistance to flow. By introducing itaconic acid (PEA-BIO-3), the viscosity significantly increased due to the bifunctional structure of itaconic acid. Instead of one carboxylic group (acrylic acid), two carboxylic groups of itaconic acid participate in cross-linking reactions. There is a significant change in the color of synthetic and bio-based polyester-acrylate resins. Synthetic resin PEA resulted in 3 Gardner, while bio-based resins PEA-BIO-1 and PEA-BIO-2 resulted in 5 and 5.5 Gardner, respectively (Figure S2). The properties of the synthetic and bio-based polyester-acrylate resins are summarized in Table 3.

**Figure 3:**
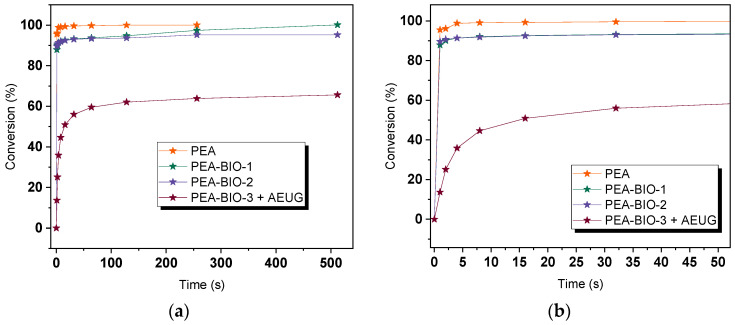
(**a**) Monitoring the acrylate conversion of synthetic (PEA) and bio-based resins (PEA-BIO-1, PEA-BIO-2 and PEA-BIO-3 + AEUG) upon irradiation with 405 nm, (**b**) the irradiation time between 0 and 50 s.

Photocurable resin formulations were then prepared by mixing the newly synthetized polyester resins with 3 wt.% of phenylbis(2,4,6-trimethylbenzoyl) phosphine oxide (Irgacure 819) as a radical photoinitiator. The cure kinetics was studied by FTIR spectroscopy in transmission mode, and the C=C-H stretching band of the acrylate (1619 and 1634 cm^−1^) and vinyl groups (1637 cm^−1^) was monitored upon exposure at 405 nm (Figure 3). The results show that the introduction of bio-based alcohols in the polyester structure slows down the reaction kinetics. For the synthetic PEA, full conversion of the acrylate groups was observed after 128 s of light exposure. In contrast, the bio-based resins PEA-BIO-1 and PEA-BIO-2 reached a conversion of 100% and 95% after 512 s of light exposure, respectively. Along with a significant reduction in viscosity, we expected that the addition of the low-molecular-weight reactive diluent AEUG accelerates the cure kinetics of PEA-BIO-3. However, the results clearly show that the replacement of acrylic acid with the less reactive itaconic acid considerably slows down the curing reactions and the functional group conversion only amounts to 65% after 512 s of light exposure. The observed results can be explained by the lower reactivity of bio-based monomers which slow down the polymerization process compared to petroleum-based monomers due to their different chemical structures. Resin PEA-BIO-3 is itaconic acid modified, while PEA, PEA-BIO-1 and PEA-BIO-2 are acrylic acid-modified unsaturated polyester resin. The difference in polymerization kinetics between acrylic- and itaconic-modified unsaturated polyester resins can be affected by chemical structure, functional groups and steric hindrance. The acrylic acid has a linear structure with one carbonyl group, while itaconic acid has a cyclic structure with two carboxyl groups. One ester group of acrylic acid easily undergoes polymerization reaction, while itaconic acid has additional functional groups such as carbon double bonds. The combined steric and electronic effects are expected to slow down the propagation of the chain-growth reaction [57].

### 3.2. Additive Manufacturing

**Figure 4:**
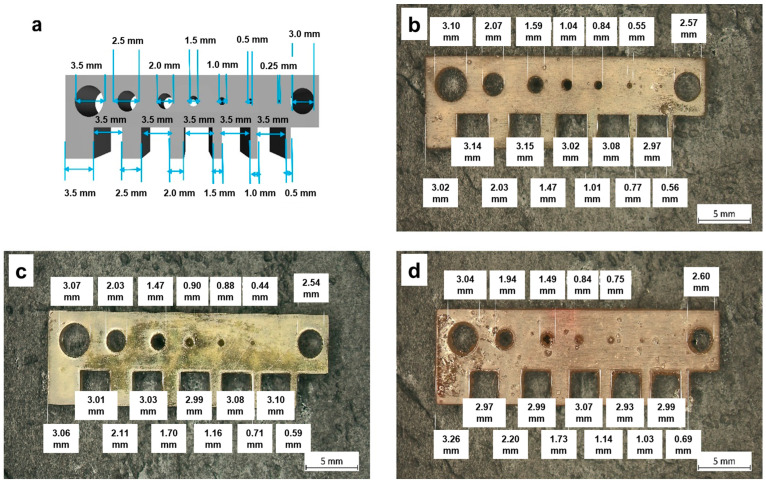
(**a**) CAD data of a comb-like test structure and DLP 3D-printed objects using different resins: (**b**) PEA, (**c**) PEA-BIO-1, (**d**) PEA-BIO-2.

To evaluate the printability of the resins and the resolution of the DLP 3D-printed objects, the resins were cured with a commercial Anycubic Photo Mono printer in layers of 100 µm with a cure time of 10 s per layer for resin PEA, 15 s per layer for resin PEA-BIO-1 and 17 s per layer for resin PEA-BIO-2. As can be seen from Figure 4, the printed specimens showed a resolution in the millimeter range. A decrease in resolution was observed when the content of bio-based alternatives was higher, which is related to their slower cure kinetics.

Due to the slow polymerization reaction and high viscosity value, the printing with PEA-BIO-3 was carried out with another printer (Doppio), which enabled printing at higher light intensity (30 mW/cm^2^) and the vat was heated at 50 °C. However, the printed specimens were full of bubbles and required a high curing time >30 s per layer (Figure S4 in the Supporting Information). Therefore, it was not possible to print specimens for resolution experiments and subsequently for thermomechanical and mechanical testing.

### 3.3. Mechanical and Thermomechanical Properties

To study the influence of the structure of the monomers on mechanical and thermomechanical properties of the related photopolymer networks, DMA and tensile tests were performed on DLP 3D-printed test specimens. In Table 4, the thermomechanical and mechanical properties of synthetic resin PEA are compared to bio-based resins PEA-BIO-1 and PEA-BIO-2.

**Figure 5:**
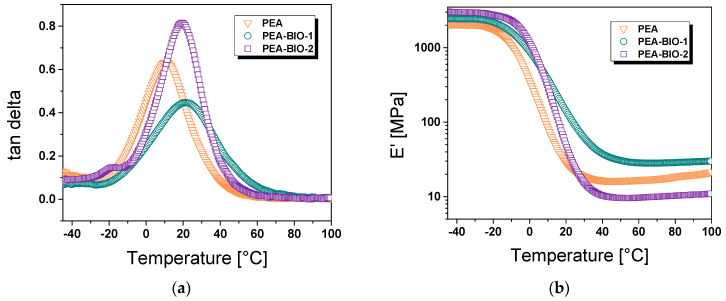
(**a**) Loss factor (*tan δ*) and (**b**) storage modulus (*E*’) versus temperature as obtained from DMA measurements of synthetic and bio-based resins.

Figure 4 shows the DMA data of the cured resins. The results show that the *T_g_* values increase from 10 to 19 and 21 °C by introducing sebacic and succinic acids, respectively. The increase in the *T_g_* may be caused by shorter-chain dicarboxylic succinic acid, whose incorporation may lead to a more rigid polymer. Due to the higher *T_g_* of PEA-BIO-1 compared to the synthetic counterpart PEA, its storage modulus (*E′*) at 23 °C was significantly higher (23.2 versus 103.7 MPa). Interestingly, PEA-BIO-2 having a *T_g_* in a similar range shows a much lower *E′* at 23 °C (30.7 MPa). This can be explained by an incomplete conversion of the resins during curing as an additional peak is observed in the *tan* delta curve at −19 °C. The unreacted polyester resin might act as plasticizer and lowers the stiffness of the related network, which is also observed in the stress–strain curves (Figure 5).

**Figure 6:**
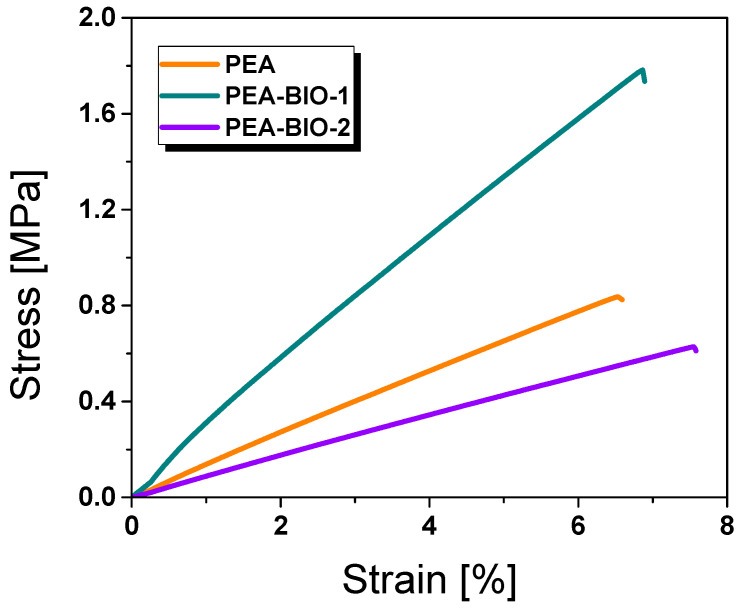
Stress–strain curves of photocured synthetic and bio-based polyester resins.

Along with the thermomechanical properties, the mechanical performance of the 3D-printed test specimens was tested and the stress–strain curves are provided in Figure 6. The synthetic resin and the bio-based ones have a similar elongation at break ranging between 6.5 and 7.5%. The stiffer PEA-BIO-1 network yields a higher tensile strength (1.73 MPa), while PEA and PEA-BIO-2 give comparable tensile strength values of 0.82 and 0.61 MPa, respectively. As can be seen from Figure 6, the tensile stress significantly increases after the incorporation of isosorbide into the polymer structure. Due to the rigid and symmetrical structure of isosorbide, which leads to strong intermolecular interactions and packing within the polymer matrix, properties can result in improved strength and stiffness. For the resin PEA-BIO-2, itaconic acid was introduced instead of acrylic acid. Acrylic-modified unsaturated polyester resins often show improved tensile strength due to acrylic functionalities which lead to intermolecular interactions and crosslinking within the polymer matrix, yielding materials with higher stiffness and strength. On the other hand, itaconic acid-modified unsaturated polyester resins can also cause increased tensile strength, albeit to a lesser extent than acrylic modification [41,58].

From the results, it can be seen that PEA-BIO-1 is an interesting alternative polyester resin for DLP 3D printing as it combines a higher content of bio-derived building blocks with decent printability and mechanical properties, which are superior to the purely petroleum-based counterpart PEA.

## 4. Conclusions

In this research, the focus was on the synthesis and characterization of DLP 3D-printable bio-based polyester resins. Using the commercially available resin PEA as a petroleum-based benchmark, monomeric building blocks were step-wise replaced with bio-based alternatives. For PEA-BIO-1, 1,6-hexanediol was substituted with bio-based 1,3-propanediol and adipic acid was replaced with bio-based succinic and sebacic acid. In the synthesis of PEA-BIO-2, the bio-based isosorbide was additionally introduced instead of cyclohexane dimethanol. Finally, for PEA-BIO-3, having the highest content of bio-based precursors, itaconic acid was additionally used instead of acrylic acid. FTIR data revealed that cure kinetics decreases with rising content of bio-based monomers. PEA-BIO-1 and BIO-2 were easily processable by DLP 3D printing, requiring a build speed of 15 to 17 s per layer (100 µm layer thickness). Due to the slow polymerization kinetics and high viscosity, PEA-BIO-3 required the addition of a bio-based reactive diluent. Although printing was carried out at a significantly higher light intensity, longer irradiation time and elevated temperature, the quality of the printed samples was inferior. Thus, no samples were printed for testing mechanical and thermomechanical properties. Synthetic resin PEA showed the lowest *T_g_* value of 10 °C, while the *T_g_* of the two bio-based resins PEA-BIO-1 and BIO-2 amounted to 19 and 21 °C, respectively. Among the studied resins, PEA-BIO-1 exhibited the highest storage modulus at 23 °C and exhibited the highest tensile strength (1.73 MPa). Based on all the results, it can be concluded that the synthesized and characterized bio-based polyester resins with reactive acrylate groups showed a great ability for additive manufacturing materials by vat photopolymerization, which was demonstrated by successful printing of tensile and DMA specimens as well as comb test structures. The presented progress enables the processing of resins with high bio-based content by using vat photopolymerization 3D printing and, thus, increases the use of sustainable and environmentally friendly materials in future applications such as soft active devices or multi-material structures [59,60].

## Figures and Tables

**Table 1:** Printing parameters.

	PEA	PEA-BIO-2	PEA-BIO-3
Time of three bottom exposure layers:	13 s	20 s	24 s
Exposure time:	10 s	15 s	17 s

**Table 2:** Used monomers in the synthesis of synthetic and bio-based resins.

PEA	PEA-BIO-1	PEA-BIO-2	PEA-BIO-3
cyclohexanedimethanol	cyclohexanedimethanol	isosorbide	isosorbide
1,6-hexanediol	1,3-propanediol	1,3-propanediol	1,3-propanediol
adipic acid	sebacic acid	sebacic acid	sebacic acid
	succinic acid	succinic acid	succinic acid
phtalic acid anhydride	phtalic acid anhydride	phtalic acid anhydride	phtalic acid anhydride
acrylic acid	acrylic acid	acrylic acid	itaconic acid
0% bio-based	40 wt.% bio-based	60 wt.% bio-based	95 wt.% bio-based

**Table 3:** Properties of synthetic and bio-based polyester-acrylate.

	PEA	PEA-BIO-1	PEA-BIO-2	PEA-BIO-3
Acid number (mg KOH/g)	5.0	9.0	13.9	83.4
Viscosity 23 °C (Pa·s)	2.31	0.27	0.96	91.78
Color (Gardner)	3	5	5.5	N/A (turbid)
Molecular weight (g/mol)	1543	936	665	1001

**Table 4:** Thermomechanical and mechanical properties of photocured synthetic and bio-based resins.

Formulation	*T_g_* (°C)	*E*′ at 23 °C (MPa)	σ (MPa)	ε (%)
PEA	10	23.2	0.82 ± 0.07	6.59 ± 0.62
PEA-BIO-1	21	103.7	1.73 ± 0.23	6.89 ± 0.69
PEA-BIO-2	19	30.7	0.61 ± 0.03	7.60 ± 0.52

## Data Availability

The raw data supporting the conclusions of this article will be made available by the authors on request.

## Supplementary Materials

The following supporting information can be downloaded at https://www.mdpi.com/article/10.3390/ma17081890/s1: Figure S1: ^1^H-NMR spectra of acrylic modified polyester resins: (a) PEA, (b) PEA-BIO-1, (c) PEA-BIO-2, (d) PEA-BIO-3; Figure S2: Appearance of synthetic and bio-based polyester acrylates; Figure S3: GPC data for (a) PEA, (b) PEA-BIO-1, (c) PEA-BIO-2 and (d) PEA-BIO-3; Figure S4: DLP 3D-printed specimens from PEA-BIO-3 resin diluted with AEUG (mass ratio was 1:1). Figure S5: Schematic representation of polyester synthesis.
