# Supplementary material for: Synthesis of Bio-Based Polyester Resins for Vat Photopolymerization 3D Printing
Source: Materials (Basel). 2024 Apr 19;17(8):1890. doi: 10.3390/ma17081890 (PMC11051430; doi:10.3390/ma17081890)
Supplement: Supplementary file 1 [file materials-17-01890-s001.zip › materials-2925578-supplementary.pdf]

materials

Supplementary Materials

Synthesis of Bio-Based Polyester Resins for Vat Photopolymerization 3D Printing

Ines Cazin [1], Martin Ocepek [2], Janez Kecelj [2], Aleš Stanislav Stražar [2] and Sandra Schlögl [1,*]

[1] Polymer Competence Center Leoben GmbH, Sauraugasse 1, A-8700 Leoben, Austria; ines.cazin@pccl.at
[2] Helios Resins, Količevo 65, 1230 Domžale, Slovenia; martin.ocepek@resinshelios.com (M.O.); janez.kecelj@resinshelios.com (J.K.); alesstanislav.strazar@resinshelios.com (A.S.S.)
* Correspondence: sandra.schloegl@pccl.at

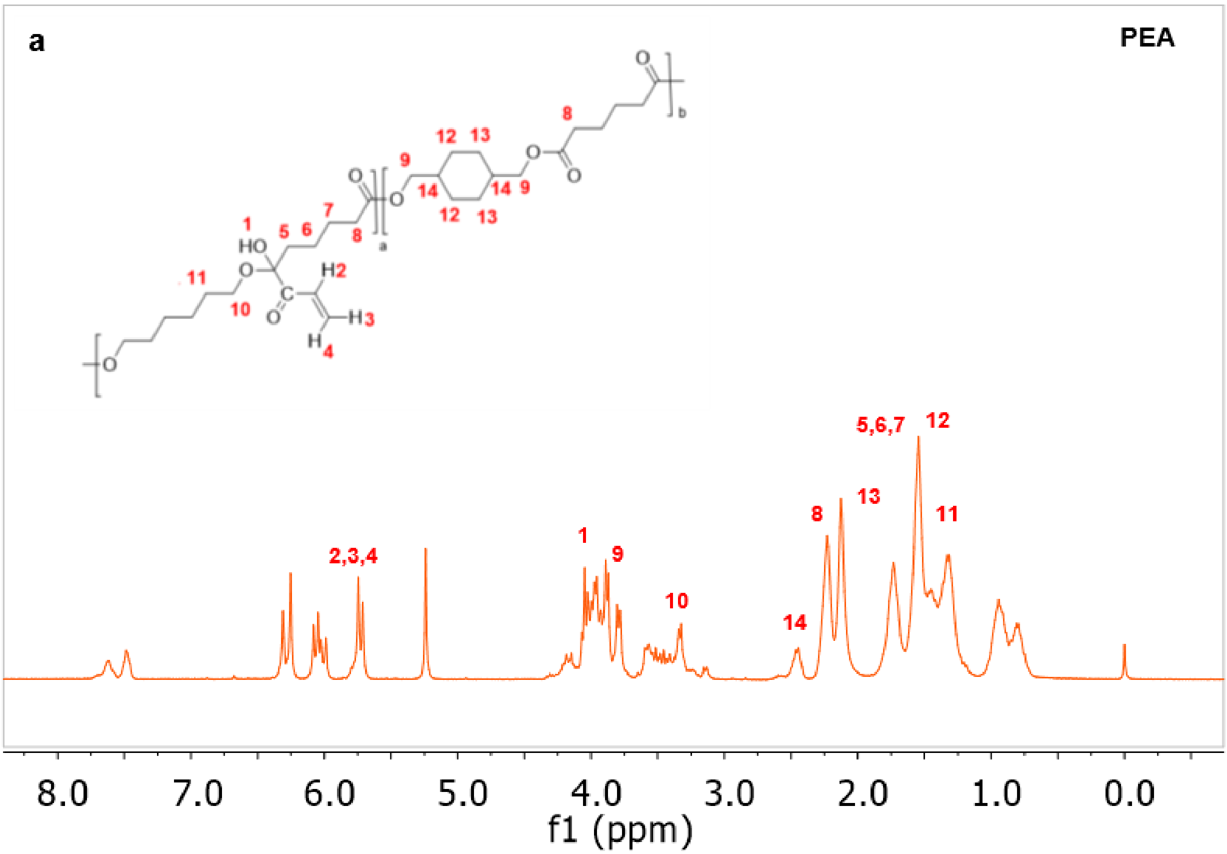

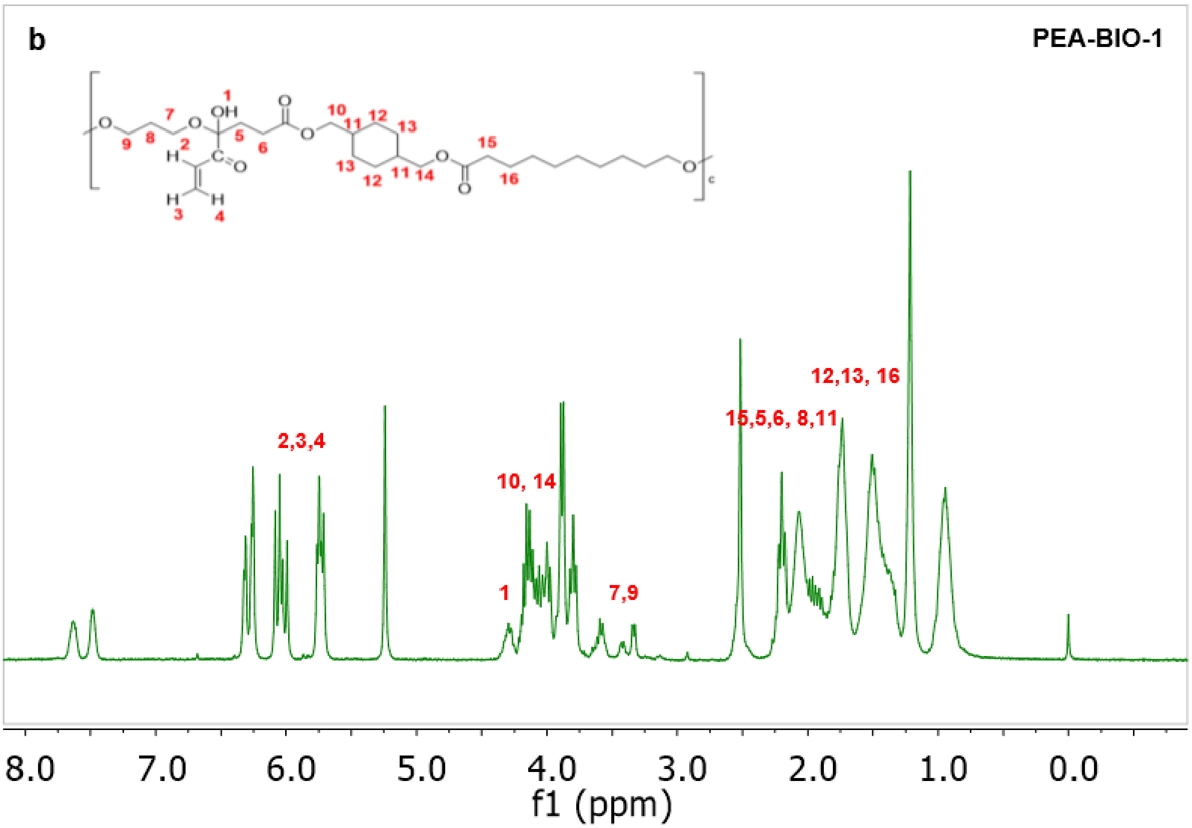
b
PEA-BIO-1
2,3,4
1
10, 14
7,9
15,5,6, 8,11
12,13, 16
8.0
7.0
6.0
5.0
4.0
3.0
2.0
1.0
0.0
f1 (ppm)

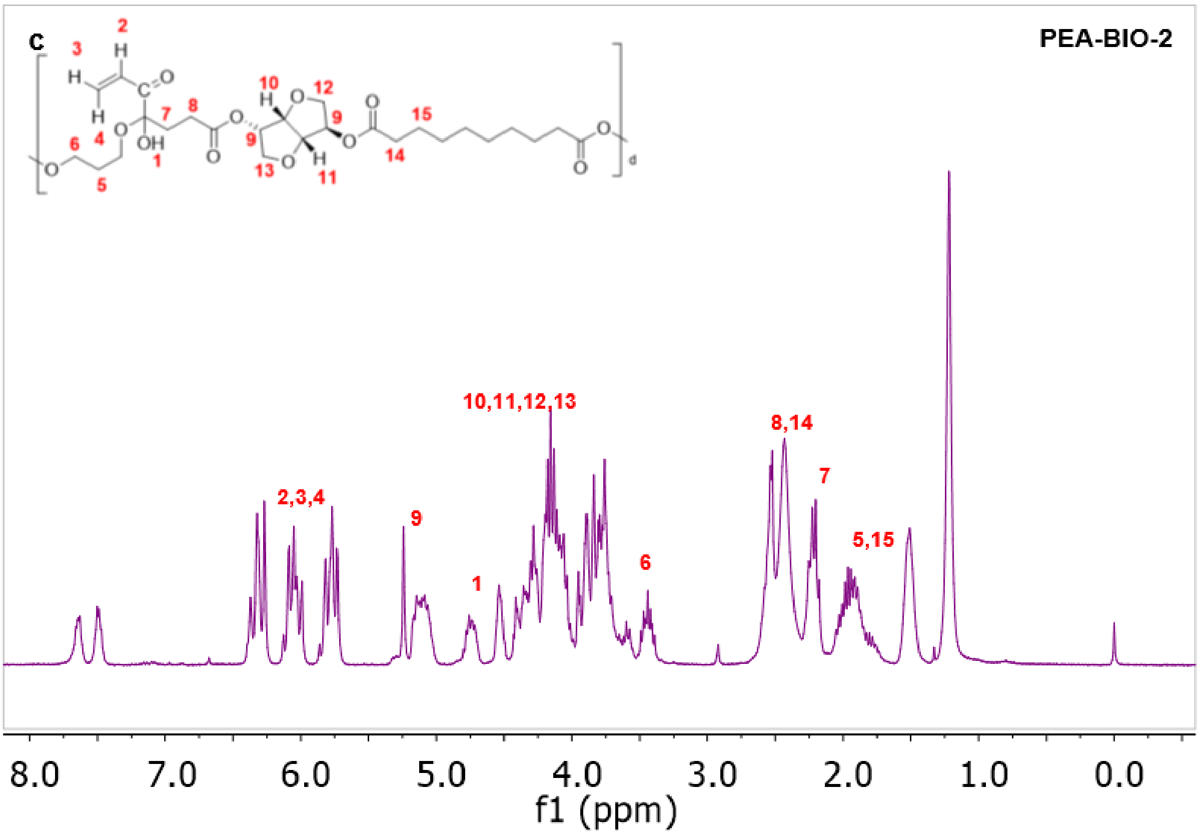
c
PEA-BIO-2
2,3,4
9
1
10,11,12,13
6
8,14
7
5,15
8.0
7.0
6.0
5.0
4.0
3.0
2.0
1.0
0.0
f1 (ppm)

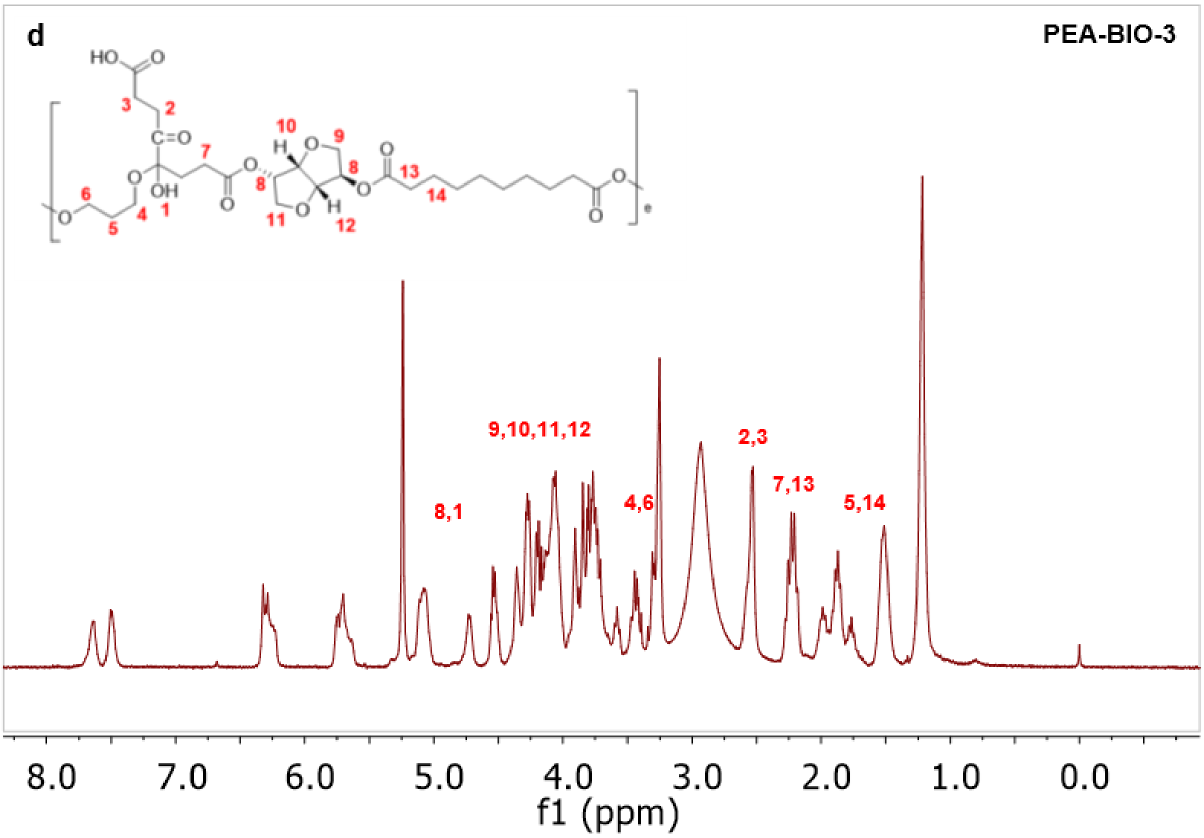

Figure S1. ^{1}H-NMR spectra of acrylic modified polyester resins: (a) PEA, (b) PEA-BIO-1, (c) PEA-BIO-2, (d) PEA-BIO-3.

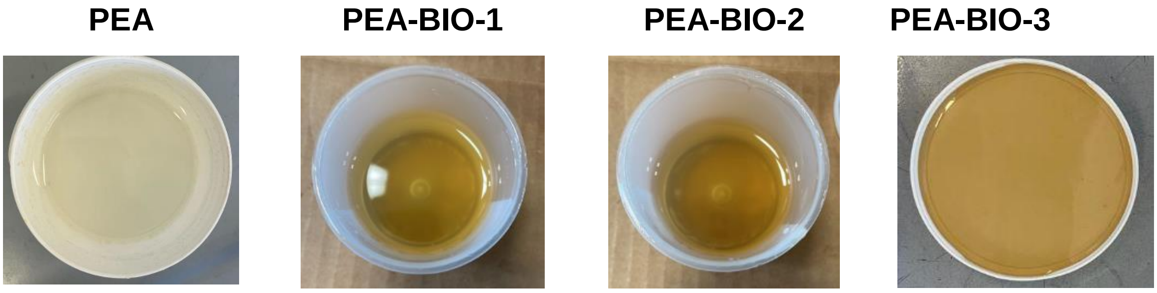

Figure S2. Appearance of synthetic and bio-based polyester acrylates.

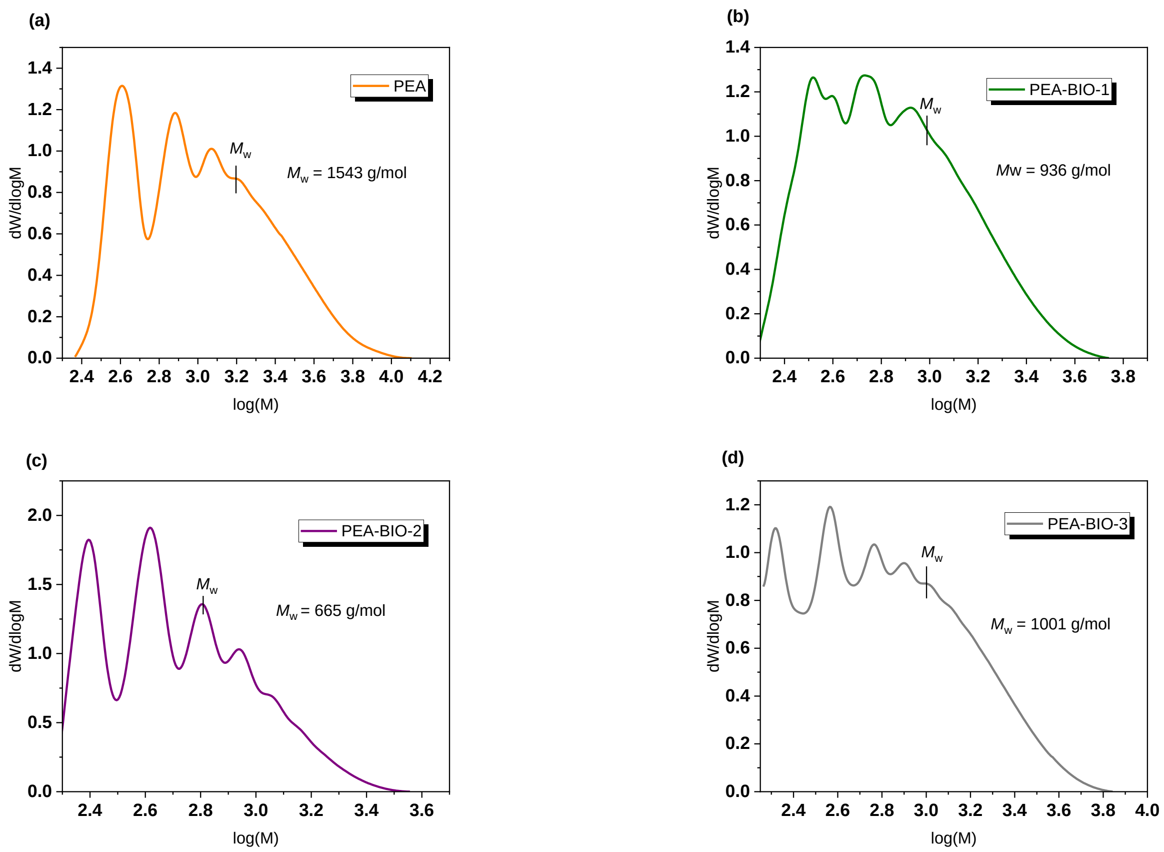

Figure S3. GPC data for (a) PEA, (b) PEA-BIO-1, (c) PEA-BIO-2 and (d) PEA-BIO-3.

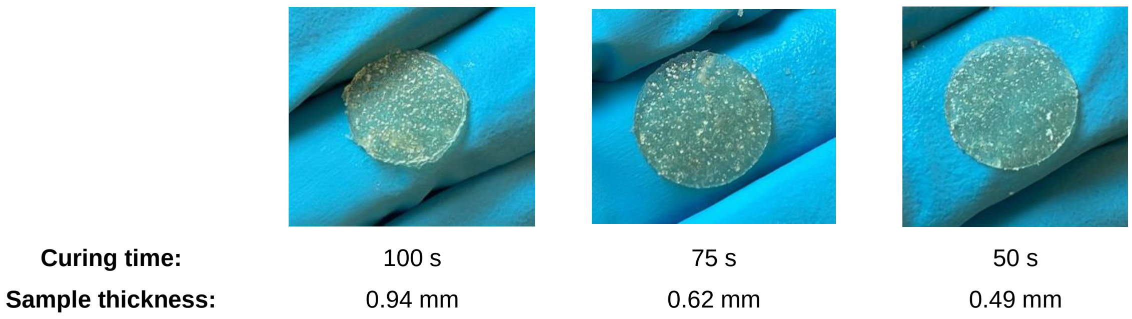

Figure S4. DLP 3D-printed specimens from PEA-BIO-3 resin diluted with AEUG (mass ratio was 1:1).

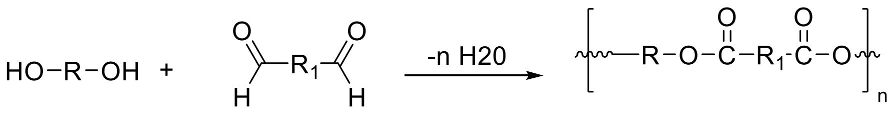

Figure S5. Schematic representation of polyester synthesis.
